# Ultra-Fast Charge Transfer in P3HT Composites Using the Core Hole Clock Technique

**DOI:** 10.3390/nano15060433

**Published:** 2025-03-12

**Authors:** Yan Li, Xiaoyu Hao, Xiongbai Cao, Tingting Wang, Haolong Fan, Lingtao Zhan, Zhenru Zhou, Huixia Yang, Quanzhen Zhang, Roberto Costantini, Cesare Grazioli, Teng Zhang, Yeliang Wang

**Affiliations:** 1 School of Integrated Circuits and Electronics & Yangtze Delta Region Academy, Beijing Institute of Technology (BIT), Beijing 100081, China; li@bit.edu.cn (Y.L.); haoxy@bit.edu.cn (X.H.); 1120210142@bit.edu.cn (X.C.); tingting.wang@bit.edu.cn (T.W.); 3120231569@bit.edu.cn (H.F.); zhanlingtao02@163.com (L.Z.); zzr2743998090@163.com (Z.Z.); yanghuixia@bit.edu.cn (H.Y.); quanzhen.zhang@bit.edu.cn (Q.Z.); 2 CNR—Istituto Officina dei Materiali (IOM), S.S. 14 km 163.5, 34149 Trieste, Italygrazioli@iom.cnr.it (C.G.); 3 Dipartimento di Fisica, Università di Trieste, Via Valerio 2, 34127 Trieste, Italy

**Keywords:** charge transfer, core hole clock, interfacial electronic coupling, poly(3-hexylthiophene)

## Abstract

Charge transfer dynamics fundamentally influence energy conversion efficiency in excited electronic states, directly impacting photoelectric conversion, molecular electronics, and catalysis. The core hole clock (CHC) technique enables the precise measurement of interfacial charge transfer time, providing insights into the electronic structure and dynamics of organic and inorganic coupled systems. Among these materials, poly(3-hexylthiophene) (P3HT), a p-type semiconductor known for its high charge mobility, serves as an ideal model for charge transfer studies. This review discusses recent advancements in understanding charge transfer dynamics in P3HT-based composites through the application of the CHC technique. The studies are categorized into two main areas: (1) P3HT combined with carbon-based nanomaterials and (2) P3HT combined with 2D materials. These findings highlight the effectiveness of the CHC technique in probing interfacial charge transfer and emphasize the critical role of nanomaterial interfaces in modulating charge transfer, which is essential for advancing organic electronic devices and energy conversion systems.

## 1. Introduction

Ultrafast charge transfer describes the rapid movement of electrons between molecules, atoms, or regions of a material within an extremely short time scale, typically from femtoseconds to picoseconds. In particular, light-induced interfacial electron transfer is fundamental to key research areas such as photoelectric conversion [1,2], photocatalysis [3,4], solar cells [5], and molecular electronics [6,7,8,9,10]. Charge transfer is a critical process that governs fundamental interactions in electronically excited states [9,11,12,13,14,15]. Interfacial electron transfer is crucial in the processes of adsorption, desorption, and dissociation reactions induced by electronic excitations. It also plays a significant role in the dynamic response of a substrate to excitations localized on an adsorbate, such as vibrational damping and screening effects [16]. Charge transfer processes at these interfaces—such as charge injection/extraction in organic light-emitting diodes (OLEDs), and electron transport across metal–molecule junctions—are crucial for optimizing device performance [17].

Therefore, charge transfer is a very worthwhile research subject. In fields such as photovoltaics and catalysis, the charge transfer time often determines the final energy conversion efficiency. For example, excessively slow charge transfer may lead to carrier recombination and reduce efficiency [18].

To study charge transfer, time-resolved spectroscopic techniques are among the most effective tools, as they provide direct insight into the ultrafast dynamics that govern charge motion within materials. The processes are studied after the illumination of a material, which can induce changes in the electronic states of a sample. This phenomenon is usually referred to as “pumping”, and the excitation source is called the “pump”. The sample response is then analyzed using two-dimensional correlation methods for a correlation map between the spectral features as a function of time. With the help of ultrafast pulsed lasers, it is possible to study processes that occur on time scales less than femtoseconds, while in the long-time scale they can extend up to picoseconds. 

Often this excitation is combined with a “probe” excitation in the so-called “pump–probe” experiment, where two ultrafast pulses are used to study dynamic processes: the pump pulse excites the sample, initiating a process such as charge transfer, electron excitation, or structural change; the probe pulse, delayed by a controlled time interval, measures the system’s response by monitoring changes in properties, such as absorption, reflection, or luminescence. In these techniques, temporal resolution is determined by the cross-correlation of the pump and probe pulses, while signal intensity depends on the system’s maximum excitation density [19].

### 1.1. Time-Resolved Photoemission Spectroscopy (TRPES)

TRPES, an extension of conventional photoemission spectroscopy, is among the most well-established techniques for investigating charge transfer [20]. In this approach, a pump pulse excites the system, promoting an electron to a higher energy state. A time-delayed probe pulse tracks the system’s evolution by detecting variations in electron emission from the sample. This technique has been widely employed to explore charge injection in dye-sensitized solar cells [21,22], electron transfer in donor–acceptor systems [23,24], and hot electron dynamics at noble metal surfaces [25,26]. The possibility of using attosecond light pulses in pump–probe experiments has recently been demonstrated; these experiments open possibilities for pump–probe experiments with sub-femtosecond resolutions. These experiments are limited to the study of small molecules [27,28], but the hope is that this technique can be extended to more complex systems and to materials science.

### 1.2. Transient Absorption Spectroscopy (TAS)

TAS is an extension of absorption spectroscopy and builds on the pump–probe concept by tracking absorption or transmittance changes in the sample over time [29,30]. If the process under study is slow, then the signal can be obtained with a continuous non-pulsed probe beam and repeated conventional spectrophotometric techniques. In contrast, for ultrafast processes, femtosecond transient absorption spectroscopy (fs-TAS) is used. The fs-TAS is a powerful tool for mapping the electron transfer path to study the dynamics of photogenerated charge carriers: by extracting decay features from the spectra, charge carrier quenching paths and their lifetimes can be simulated on femtosecond and picosecond time scales. This technique is widely applied to the study of charge transfer in photocatalysis in semiconductors [29,31], metal-organic frameworks [32,33], and organic photovoltaic blends [34]. Recently, attosecond transient absorption spectroscopy achieved a temporal resolution below 400 attoseconds (as) at the argon L_2,3_ edges [34], paving the way for attosecond-scale studies in materials science.

### 1.3. Time-Resolved Photoluminescence (TRPL)

Time-resolved photoluminescence (TRPL) measures the temporal decay of photoluminescence following photoexcitation, which is closely tied to recombination dynamics and charge separation. Steady-state photoluminescence (PL) spectroscopy is frequently employed to assess the impact of charge transfer, as a reduction in the PL signal, known as quenching, often indicates the occurrence of charge transfer. Achieving time-resolved measurements depends on the specific experimental requirements, with various approaches available to balance sensitivity and temporal resolution [35]. Typically, TRPL offers insights into long-lived states. Traditional TRPL setups operate with picosecond to nanosecond time resolution, contingent on the detector and excitation source. This is sufficient for studying slower processes, such as exciton recombination and charge separation, complementing the short-time resolution of pump–probe measurements.

While the time-resolved techniques discussed above provide valuable insights into charge transfer dynamics, they primarily rely on capturing temporal changes following an external excitation, which requires a pulsed light source, and introduces certain limitations. For example, although TRPES achieves femtosecond to picosecond resolution, it requires synchronized ultrafast lasers and synchrotron/X-ray free-electron laser sources, significantly increasing experimental complexity.

In contrast, since the early 1990s, the so-called core hole clock (CHC) technique [36,37,38,39,40,41,42] enabled investigations at time scales even shorter than 1 fs. Unlike conventional time-resolved methods, CHC does not require femtosecond (fs) or picosecond (ps) laser pulses. Instead, it utilizes the intrinsic lifetime of core holes as a natural clock to measure ultrafast charge transfer dynamics at interfaces. This approach relies on using the decay time of the intermediate (core hole) state as an internal clock—hence the name “core hole clock”. Due to its capability of probing ultrafast charge transfer processes in complex environments such as buried interfaces or within complex molecular architectures, the CHC technique has emerged as a key tool for investigating charge transfer in optoelectronic and catalytic materials [16,43,44,45,46,47]. This review provides an in-depth analysis of the applications of the CHC technique in studying charge transfer within molecular systems as well as at interfaces. The focus of the present review is on integrating CHC with structural and morphological analyses for a more comprehensive understanding of the phenomenon.

**Figure 1:**
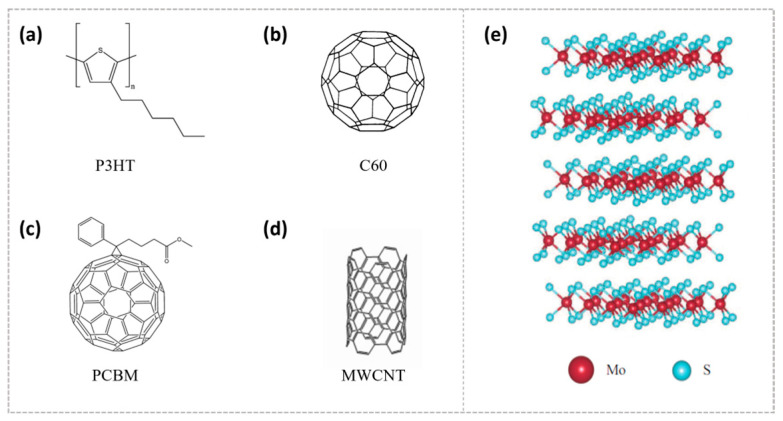
The chemical structures are as follows: (**a**) poly(3-hexylthiophene), known as P3HT; (**b**) C60; (**c**) [6,6]-phenyl-C61-butyric acid methyl ester (PCBM); and (**d**) MWCNT. (**e**) The honeycomb structure of MoS_2_ consists of stacked S–Mo–S units. (**a**,**c**) Reprinted with permission from Ref. [48] from the American Chemical Society, copyright 2016; (**b**) Reprinted with permission from Ref. [61] from the American Chemical Society, copyright 1991; (**d**) Reprinted with permission from Ref. [62] from Elsevier, copyright 2021; (**e**) Reprinted with permission from Ref. [63] from the American Chemical Society, copyright 2014.

Poly(3-hexylthiophene) (P3HT, Figure 1a) has emerged as an ideal p-type semiconductor material for constructing heterojunction devices due to its suitable band gap of 1.9–2.0 eV, excellent charge transfer properties, and solution processability [48,49,50]. The P3HT polymer and carbon-based nanomaterial composites and two-dimensional materials have been widely explored in heterojunction blends [51,52,53,54,55,56,57,58,59,60]. Among these, the composite systems of fullerene (C60, Figure 1b) and its derivative PCBM (Figure 1c) with P3HT, as well as graphene and multi-walled carbon nanotubes (MWCNT, Figure 1d) with P3HT, have demonstrated significant potential for applications. The van der Waals heterojunctions formed between P3HT and two-dimensional materials exhibit significant promise for device applications, owing to the quantum confinement effect of 2D materials, their large absorption cross-section, excellent mechanical flexibility, and tunable synthesis properties of organic semiconductor polymers [51].

Currently, research focuses on the interfacial charge transfer phenomena between organic semiconductor polymer layers, two-dimensional materials, and carbon-based nanomaterials [64,65]. However, due to the limitations of laser bandwidth and the time delay between the pump and probe lasers, this method can only detect ultrafast processes on the femtosecond (10^−15^ s) timescale [66]. With recent advances in laser technology and high-harmonic generation, transient absorption techniques using broadband pulses as probes in the ultraviolet-visible and soft X-ray ranges can achieve sub-femtosecond time resolution [67].

In the review, we present an alternative approach—the CHC technique—which enables the effective detection of charge transfer dynamics on attosecond (10^−18^ s) or sub-femtosecond timescales [68]. We summarize recent studies on charge transfer dynamics in P3HT composites using the CHC technique, emphasizing its significance in understanding interfacial charge transfer processes.

## 2. The Core Hole Clock Technique

Advancements in synchrotron radiation facilities have enabled the production of tunable, continuous photon energies, facilitating techniques such as resonant photoelectron spectroscopy and resonant Auger spectroscopy [69,70,71,72] These developments have made the CHC technique feasible, allowing for the investigation of ultrafast electron dynamics at interfaces. The CHC technique was proposed and developed in the 1990s, based on the approach of using synchrotron radiation to study and analyze charge transfer dynamics among various materials. This method leverages the core hole lifetime as an internal clock, utilizing the excitation and decay of core electrons to measure ultrafast charge transfer times at interfaces [36]. It is particularly useful for investigating charge transfer dynamics at organic–inorganic interfaces [73]. The core hole lifetime enables temporal resolution of charge transfer dynamics on femtosecond to attosecond timescales. In CHC technique, core electron excitation creates a transient state where charge transfer to the substrate can compete with core hole decay. If electron transfer occurs before Auger relaxation (occurring within ~1 fs), the Auger emission characteristic of the adsorbate is attenuated. The systematic comparison of Auger spectra under resonant and non-resonant excitation conditions allows for the quantitative determination of charge transfer timescales. A key advantage of CHC is that it inherently provides atomic specificity, with electronic transitions involving core electrons that can be selectively attributed to specific chemical species, thereby providing both temporal resolution and chemical sensitivity.

The core-level hole clock spectrum is valid for estimating the charge transfer time only when the relationship between the charge transfer time ($\tau_{ct}$) and the core hole lifetime ($\tau_{core}$) satisfies 0.1 $\times \tau_{core}$ ≤ $\tau_{ct}$ ≤ 10 × $\tau_{core}$. When $\tau_{ct}$ ≤ $\tau_{core}$, the measured results correspond to the final state data after charge transfer to the substrate’s continuum states. The time of charge transfer can be calculated using the CHC equation:

(1) $$\tau_{ct}=\frac{I_{spectator}+I_{participator}}{I_{Auger}}\times \tau_{core}$$

Here, $\;\tau_{core}$ denotes the core hole lifetime, $I_{Auger}$ represents the intensity of the normal Auger component, and $I_{spectator+participator}$ corresponds to the spectator and participator component [11]. The value of $\tau_{core}$ is typically taken from widely accepted references in the field. However, it can also be determined theoretically or measured experimentally through lifetime broadening in high-resolution photoemission core-level spectra [74,75]. In this work, we adopted $\tau_{core}$ = 1.27 fs, corresponding to the S1s core hole lifetime [76]. We note that although the $\tau_{core}$ value may introduce some error, the main source of inaccuracy in the CHC technique comes still from the experiment, particularly the spectral fitting procedure.

**Figure 2:**
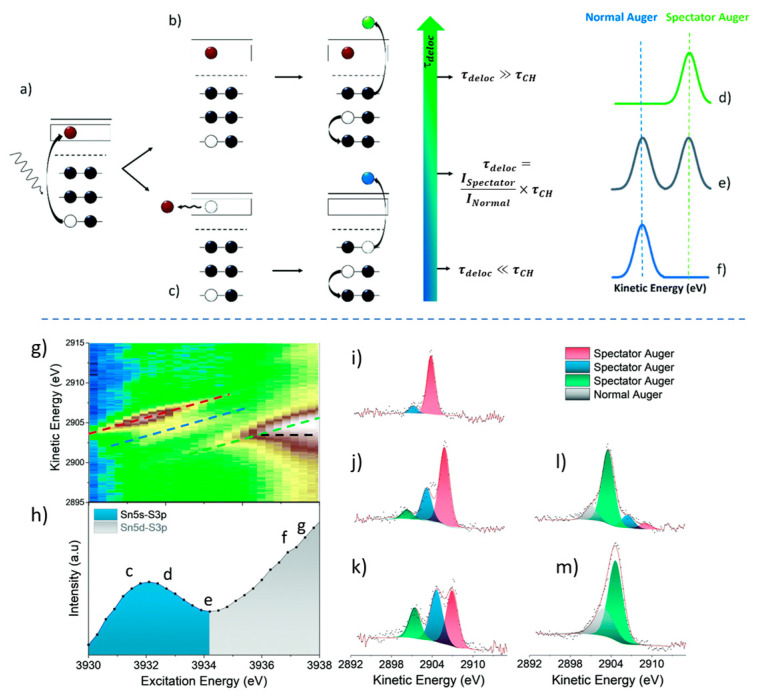
Upper part: Schematic representation of the CHC technique. Upon absorption of electromagnetic radiation, a core electron is excited to the conduction band, leaving behind a core hole (**a**). The excited electron can either remain localized in the atomic orbital within the conduction band, leading to the emission of a spectator Auger electron (**b**), or delocalize within the conduction band, resulting in the emission of a normal Auger electron (**c**). Depending on the core hole lifetime ($\tau_{CH}$) and the delocalization time of the excited electron ($\tau_{deloc}$), three distinct types of Auger spectra can be observed (**d**–**f**). Lower part: (**g**) The Sn L3 edge XAS spectra of SnS, with spectator Auger dispersion trends highlighted. (**h**) One-dimensional (1D) Auger yield spectra at the Sn L3 edge. (**i–m**) Sn L3NN Auger scans at selected photon energies, as indicated in (**h**). Reprinted with permission from Ref. [43] from The Royal Society of Chemistry, copyright 2021.

In detail, we use Figure 2 (upper part) to illustrate the basis of the method for a single-atom event. The absorption of an X-ray near the absorption edge excites an electron to an orbital in the conduction band, creating a core hole (Figure 2a). This excited state relaxes via Auger decay, where an electron from an outer shell fills the core hole, leading to the emission of an Auger electron (Figure 2b,c). The nature of the Auger emission depends on the delocalization time of the excited electron: if it remains localized longer than the core hole lifetime, a spectator Auger is emitted (Figure 2b), shifting in kinetic energy with the excitation energy due to energy conservation. If the electron delocalizes faster, a normal Auger is emitted (Figure 1c), with constant kinetic energy independent of the excitation energy. An example of this behavior is shown in the lower part of Figure 2.

What differentiates CHC is its extraordinary capacity to accurately measure the femtosecond charge transfer time scale of different orbitals within a single molecule. This makes it an invaluable tool when studying metal–organic systems, in which multiple orbitals may contribute to the charge injection into the substrate [43,77,78]. Integrating the CHC technique with complementary characterization techniques enables a more comprehensive understanding of charge transfer dynamics, which is crucial for advancing the design and optimization of new materials [44,45,79,80,81].

Separating the contributions from localization, delocalization, and non- charge transfer processes in the CHC technique can be challenging. In the interpretation of CHC data, theoretical frameworks such as density functional theory (DFT) and time-dependent DFT (TDDFT) provide critical insights into electronic structure and charge transfer pathways [43,82]. For instance, DFT calculations have been employed to map the unoccupied states involved in core excitations, while TDDFT simulations corroborate the observed sub-femtosecond charge transfer dynamics by modeling electron delocalization mechanisms at hybrid interfaces.

While CHC is effective for interfacial studies, its surface sensitivity may limit its applicability for probing bulk charge transfer processes in thicker films or materials. The CHC technique indirectly infers charge transfer times by probing the relaxation processes of core-level holes, whereas charge transfer in practical devices (e.g., carrier transport in optoelectronic devices [83]) typically depends on valence-band or conduction-band electron dynamics. Consequently, CHC technique measurements generally cannot be directly equated to the actual charge transfer times occurring during device operation. Additionally, CHC experiments typically require high-intensity X-ray sources, such as synchrotrons, whose accessibility is limited, and beamtime acquisition is highly competitive [84,85].

## 3. P3HT-Based Heterojunctions: Ultrafast Charge Transfer and Interfacial Interactions

Polythiophenes, particularly its derivative poly(3-hexylthiophene) (P3HT), are among the most notable and extensively researched semiconducting polymers. They have found applications in a wide range of devices, such as solar cells and field-effect transistors [86,87]. Understanding the occupied and unoccupied electronic structures, as well as the charge transfer kinetics of these materials, is of importance. 

This review highlights the ultrafast charge transfer in P3HT-based heterojunctions studied using the CHC technique. We first examine P3HT with carbon-based nanomaterials, followed by its combination with 2D materials.

The P3HT polymer, in combination with MWCNT and carbon-based nanomaterials like fullerene (C60 and its derivative PCBM) and graphene, has been extensively utilized in bulk heterojunction devices [88,89,90,91,92,93,94,95,96]. The interfacial charge transfer occurring between P3HT and carbon nanotubes (CNTs) is of vital importance in determining the performance of the device. Its efficiency is affected by various factors, including interface hybridization and film morphology.

**Figure 3:**
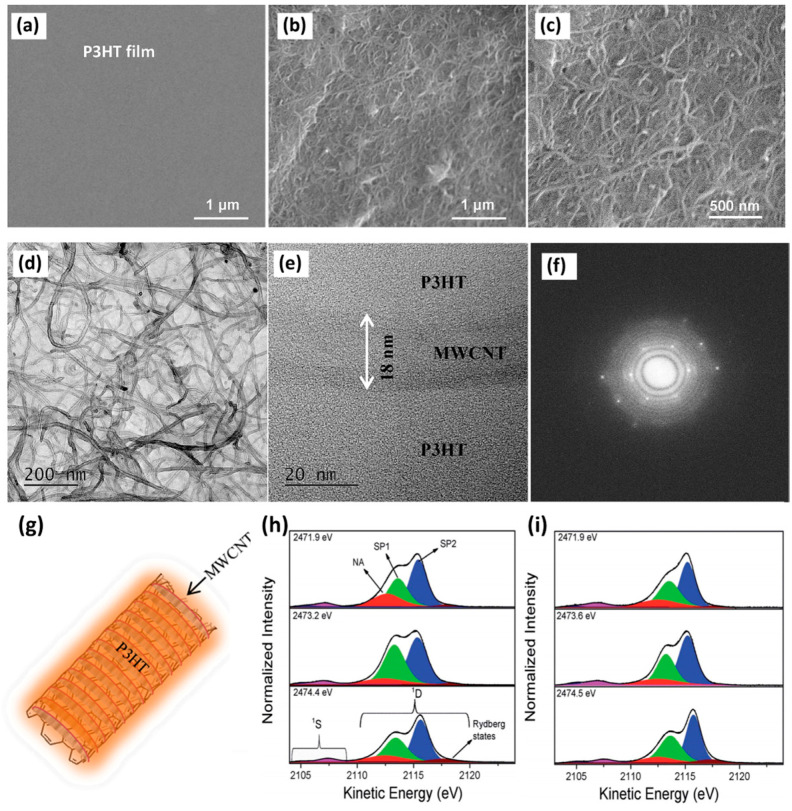
(**a**–**c**) FESEM images. (**a**) Pristine P3HT film, (**b**) P3HT/MWCNT composite, and (**c**) P3HT/MWCNT composite at a higher resolution of 500 nm. (**d**) TEM, (**e**) HRTEM, and (**f**) selected area diffraction (SAED) pattern of the composite. (**g**) A schematic representation of the P3HT/MWCNT structure. (**h**) P3HT film and (**i**) P3HT/Fe-MWCNT-5% film. The spectator and normal Auger decay channels are labeled as SP1 ($\pi^{*}$, green), SP2 ($\sigma^{*}$, blue), and NA (normal Auger channel, red). The Rydberg state is denoted by a wine color. (**a**–**g**) Reprinted with permission from Ref. [97] from Elsevier, copyright 2017; (**h**,**i**) Reprinted with permission from Ref. [56] from The Royal Society of Chemistry, copyright 2018.

Mahakul P. C. and colleagues found that MWCNTs significantly enhance the conductivity and optoelectronic properties of P3HT composites by lengthening the conjugation length of the polymer chains and strengthening interfacial interactions [97]. Figure 3a–c display the images of P3HT films and MWCNT-doped P3HT composite films obtained using field emission scanning electron microscopy (FESEM). It was observed that the MWCNTs form wavy, worm-like structures that are effectively embedded within the P3HT matrix. Figure 3d,e show the fine structural features of the composite material. There was good interface bonding between MWCNT structures and the P3HT matrix. Figure 3f displays the hexagonal crystal diffraction pattern of the composite, confirming the high crystallinity of the MWCNTs. Figure 3g shows a schematic of the P3HT/MWCNT structure.

Ultrafast charge and energy transfer at the P3HT–CNT interfaces has been previously studied through laser-excited pump–probe spectroscopy [98,99]. It has been shown that incorporating just 1% of single-walled carbon nanotubes (SWNTs) into P3HT facilitates highly efficient photon-to-charge conversion. Their findings revealed that charge transfer occurs remarkably fast, on the order of ~430 fs, while also enabling long-term charge separation at room temperature, particularly when small-diameter nanotubes are uniformly dispersed within the P3HT matrix [64]. The sub-fs time scale has also been explored by the CHC technique, showing that the electronic interaction between P3HT and Fe-MWCNT is influenced by the nature of the electronic excited states [56].

The sulfur–KL_2,3_L_2,3_ resonant Auger spectra and their deconvolution results for P3HT and P3HT/Fe-MWCNT-5% films are shown in Figure 3h,i. These experiments were performed at the French synchrotron facility SOLEIL, GALAXIES beamline, on the hard X-ray photoelectron spectroscopy (HAXPES) end station. The shift measured the kinetic energy difference between spectator features (SP1, SP2) and non-resonant (normal) Auger (NA) decay. At three different photon energies, the analysis of the SP1 spectator shift parameters of the resonant Auger spectra indicated a greater electron delocalization in P3HT/ITO compared to P3HT/Fe-MWCNT-5% for both the $\pi^{*}$ (2471.9 eV) and $\sigma^{*}$ symmetry (2474.4 eV) excited states.

Nevertheless, at the photon energy corresponding to the resonance maximum (2473.2 eV), the electron delocalization in the P3HT/Fe-MWCNT-5% nanocomposite was notably greater compared to P3HT/ITO. This suggests that the inclusion of Fe-MWCNT enhances the electron delocalization within P3HT. The addition of Fe-MWCNT substantially lessened the interchain interactions of P3HT. As a result, the interchain charge transfer time increased from 4.7 fs in the pure P3HT polymer to 6.5 fs in the P3HT/Fe-MWCNT-5% composite. The quantitative results are presented in Table 1, compared to the P3HT:PCBM blend.

**Figure 4:**
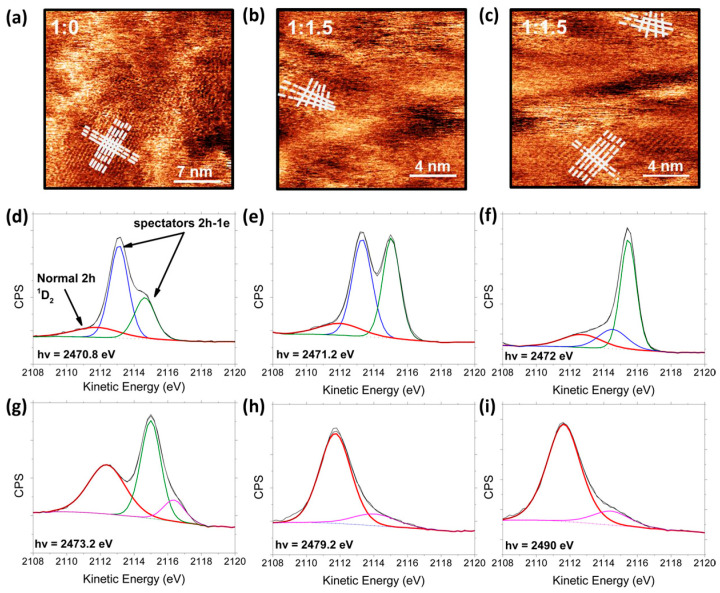
(**a**–**c**) High-resolution AFM phase images of pure P3HT films, with white lines indicating regions where the polythiophene chains diverge in the 1:1.5 P3HT:PCBM ratio. (**d**–**i**) Auger spectra for P3HT:PCBM films captured at specific photon energies within the XAS spectrum. (**a**–**c**) Reprinted with permission from Ref. [100] from the American Chemical Society, copyright 2022; (**d**–**i**) Reprinted with permission from Ref. [48] from the American Chemical Society, copyright 2016.

In organic photovoltaics, P3HT and PCBM serve as common electron donor and acceptor materials. Figure 4a–c show the high-resolution atomic force microscopy (AFM) phase images of the P3HT and 1:1.5 P3HT:PCBM blend film [100]. In the pristine P3HT film, polythiophene chains were arranged in parallel to form an obvious square lattice structure, while in the 1:1.5 P3HT:PCBM blend, this lattice structure was affected by PCBM and partially distorted, indicating the influence of the acceptor molecule (PCBM) on the polythiophene crystal domain. The charge transfer time at the S-K L_2,3_L_2,3_ edge absorption for the P3HT:PCBM blend was significantly shorter than that measured for polythiophene and related polymers that were blended with fullerene [48].

In Figure 4d–i, the resonant Auger spectra of the P3HT:PCBM blends are presented under various photon energy conditions, measured using a hemispherical electron energy analyzer with a pass energy of 20 eV. At the resonant energy points (d–g), where the excited electron remained in a high-energy state, different characteristic peaks of the Auger decay can be observed. On the other hand, at higher energy points (h,i), the Auger decay were primarily dominated by the “normal” Auger channel, with the Auger electrons having higher kinetic energy, indicating that these Auger electrons are fully delocalized. Table 1 presents that the charge transfer in this system occurs within the femtosecond time scale, with all recorded values below 9 fs, and the highest being 8.49 fs. In comparison to studied P3HT/MWCNT nanocomposites, the P3HT:PCBM blend demonstrated the fastest charge transfer time ever recorded at the S-K L_2,3_L_2,3_ edge, employing the CHC technique. This observation also supports its superior performance in devices, establishing it as one of the leading materials for bulk heterojunction (BHJ) solar cells [48].

**Figure 5:**
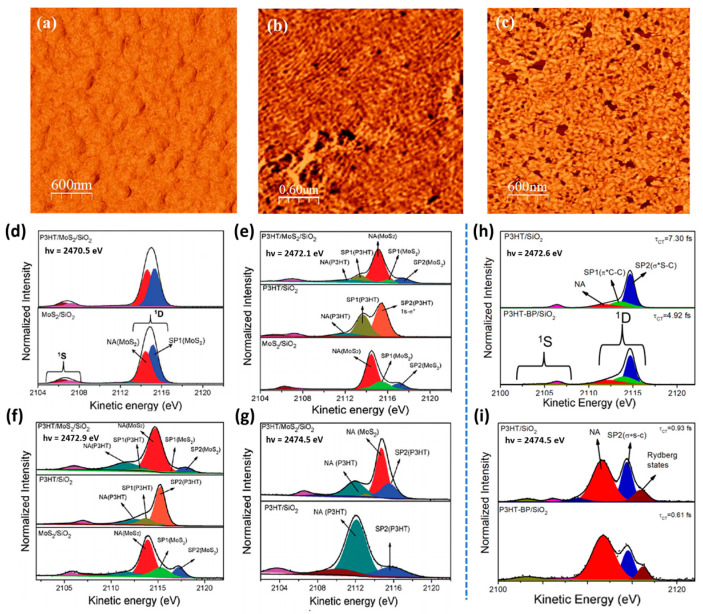
AFM phase images showing the (**a**) pristine P3HT, (**b**) P3HT/MoS_2_ (1%), and (**c**) P3HT/MoS_2_ (2%) nanocomposites, all of which are deposited onto a SiO_2_/Si substrate. (**d**–**g**) Resonant Auger spectra (S-K L_2,3_L_2,3_) and the results of their deconvolution for the MoS_2_/SiO_2_, P3HT/SiO_2_, and P3HT/MoS_2_/SiO_2_ thin films, recorded at different excitation energies. (**h**,**i**) S-K L_2,3_L_2,3_ resonant Auger spectra and their deconvolution results for the P3HT/SiO_2_ thin film (top) and P3HT-BP/SiO_2_ heterojunction (bottom), recorded at excitation energies (**h**) 2472.6 eV and (**i**) 2474.5 eV. (**a**–**c**) Reprinted with permission from Ref. [59] from the American Chemical Society, copyright 2020; (**d**–**g**) Reprinted with permission from Ref. [51] from the Owner Societies, copyright 2019; (**h**,**i**) Reprinted with permission from Ref. [101] from Elsevier, copyright 2021.

Then, we will discuss the ultrafast charge transfer in P3HT in combination with 2D materials studied using the CHC technique. With the rapid advancement of materials science, hybrid van der Waals heterostructures integrating organic polymers and two-dimensional materials have emerged as a critical strategy for enhancing carrier mobility and interfacial charge separation efficiency in optoelectronic devices, leveraging their unique interfacial engineering merits. Molybdenum disulfide (MoS_2_), with its unique optoelectronic properties, holds significant potential for use in photonics and optoelectronic devices. By combining P3HT with MoS_2_, a van der Waals-based organic/two-dimensional (2D) heterojunction can be formed. Figure 5a–c display the AFM phase images of both pristine P3HT and P3HT/MoS_2_ (1%, 2%) thin films, and MoS_2_ is a few-layered structure [59]. The content of MoS_2_ is defined by the mass percentage of MoS_2_ nanosheets relative to P3HT [59]. In Figure 5a, the pristine P3HT film exhibited a smooth, globular structure, with no evident ordered fibril formation. In contrast, in Figure 5b,c, the fibers were seen to be evenly distributed and arranged in a more orderly manner, suggesting that the addition of MoS_2_ nanosheets facilitates the development of long fibers. However, an excessive amount of MoS_2_ can lead to the polymer chains being arranged randomly. The introduction of an appropriate amount of MoS_2_ (such as 1%) can significantly improve the self-assembly and crystallization properties of P3HT chains, thereby forming an ordered long fibrous structure. In a study by Garcia-Basabe et al., sub-fs charge transfer was observed within the P3HT/MoS_2_/SiO_2_ heterojunction [51]. The reported charge transfer times were 0.34 fs for electrons excited to the S 3pz states of MoS_2_, and 0.45 fs for those excited to the π* (C-C) states of P3HT in Table 2.

The S-K L_2,3_L_2,3_ resonant Auger spectra and corresponding deconvolution results for the MoS_2_/SiO_2_, P3HT/SiO_2_, and P3HT/MoS2/SiO_2_ thin films are shown in Figure 5d–g, with data collected at varying excitation energies. For a more precise evaluation of the electron delocalization dynamics at the P3HT/MoS_2_/SiO_2_ interface, $\tau_{ct}$ was derived as defined by Equation (1), using $\tau_{core}$ = 1.27 fs (the S1s core hole lifetime). In Figure 5d, when electrons are excited to the S 3px,y state, the $\tau_{ct}$ values for the isolated MoS_2_/SiO_2_ (1.32 fs) and P3HT/MoS_2_/SiO_2_ (1.25 fs) thin films were nearly identical. Notably, no interfacial charge transfer was detected in the P3HT/MoS_2_/SiO_2_ film. This suggests that the femtosecond charge transfer observed for electrons in the S 3px,y state is mainly associated with intra-layer charge transfer processes. In Figure 5e, the $\tau_{ct}$ values for MoS_2_ species, following excitation of electrons to the S3pz state, decreased from 0.62 fs in the MoS_2_/SiO_2_ film to 0.34 fs in the P3HT/MoS_2_/SiO_2_ heterojunction. The intra-layer pathway is the primary electron delocalization route for electrons in the S3pz state within the MoS_2_/SiO_2_ thin film. Consequently, in the P3HT/MoS_2_/SiO_2_ heterojunction, a new sub-fs electron delocalization pathway from MoS_2_ to P3HT was established, reducing the $\tau_{ct}$ value by approximately 50%.

In Figure 5f, the charge transfer time for the P3HT species decreased dramatically, from 4.13 fs in the P3HT/SiO_2_ thin film to 0.45 fs in the P3HT/MoS_2_/SiO_2_ heterojunction. Similarly, the $\tau_{ct}$ for the MoS_2_ species at the same excitation energy reduced from 0.50 fs in MoS_2_/SiO_2_ to 0.20 fs in the P3HT/MoS_2_/SiO_2_ heterojunction. These observations revealed that interfacial electron transfer between P3HT and MoS_2_ occurs bidirectionally, with a more efficient transfer from P3HT to MoS_2_. In Figure 5g, analysis of the electrons excited to the $\sigma^{*}$(S-C) state indicated that both the P3HT/MoS_2_/SiO_2_ heterojunction and P3HT/SiO_2_ thin film exhibited a $\tau_{ct}$ value of approximately 0.30 fs. This suggests that for the S1s-$\sigma^{*}$(S-C) electron transition in P3HT, no interfacial charge transfer is present between P3HT and MoS_2_. The strong electronic coupling between the S3pz states of MoS_2_ and the π*(C-C) states of P3HT facilitates charge transfer at the interface of the P3HT/MoS_2_/SiO_2_ heterojunction.

In recent years, black phosphorus (BP), a member of the 2D layered materials family, has attracted significant attention due to its potential in diverse technological fields [102,103,104,105]. With a large specific surface area, excellent charge carrier mobility, and a bandgap that is adjustable with thickness, this material offers promising characteristics [102]. Heterojunctions of P3HT and BP have also been reported, showing that the $\pi^{*}$ (S-C) electronic state serves as the fastest electron delocalization route from the P3HT to the BP conduction band [101]. In Figure 5h,i, the $\tau_{ct}$ values for electrons excited into the $\pi^{*}$ (S-C) state decreased from 7.30 fs in P3HT/SiO_2_ to 4.92 fs in the P3HT-BP/SiO_2_ heterojunction. Similarly, for the S 1s-σ*(S-C) transitions, $\tau_{ct}$ shortens from 0.93 fs to 0.61 fs. This observation implies a significant electronic interaction between P3HT and BP, particularly for $\pi^{*}$ (S-C) transitions, with a reduction of approximately 40% in $\tau_{ct\;}$.

In summary, interfacial charge transfer processes are influenced by various factors, among which electronic coupling is one of the key elements [106,107,108,109]. In Table 3, we can see a comparative data table of the charge transfer times between different P3HT heterojunctions, as determined by the CHC technique. In the P3HT/MoS_2_ system, charge transfer occurs primarily via the $\pi^{*}$ (S-C) state in P3HT and the conduction band of MoS_2_, facilitated by a strong electronic interaction between the two components. Similarly, in the P3HT/BP system, efficient charge transfer can be attributed to the robust coupling between the $\pi^{*}$ (S-C) state of P3HT and the conduction band of BP. Therefore, selecting materials with complementary electronic states is crucial for maximizing charge transfer efficiency. The aforementioned material heterojunctions demonstrate how the morphology of the system influences the charge transfer dynamics, thus confirming that tailoring the interface design is especially critical for achieving the desired functionality in applications such as photovoltaic devices, sensors, LEDs, and more.

## 4. Conclusions

The CHC technique serves as an invaluable resource for examining ultrafast charge transfer dynamics, offering insights into electronic behavior within the sub-femtosecond to femtosecond timescale. This review presents a concise overview of the investigations into interfacial charge transfer between P3HT polymers and various carbon-based nanomaterials, including MWCNTs and PCBM, as well as two-dimensional materials like MoS_2_ and BP, highlighting the imperative of interface tailoring for application-specific optimization in photovoltaic devices, sensors, and light-emitting diodes (LEDs). The CHC technique coupled with advanced experimental techniques and theoretical models is crucial for enhancing our comprehension and further optimizing the performance of organic optoelectronic devices. This comprehensive, multi-scale approach will deepen our understanding of charge transfer processes in organic/heterojunctions and further advance the development of high-performance organic optoelectronic devices.

## Figures and Tables

**Table 1:** Charge transfer times ($\tau_{ct}$) in femtosecond (fs) for P3HT, P3HT:PCBM blend films [48] and P3HT/Fe-MWCNT-5 [56].

Photon Energy (eV)	$\tau_{ct}$ ***(fs)***		
	P3HT	P3HT/PCBM	P3HT/Fe-MWCNT-5% (fs)
2470.8	-	7.19	-
2471.2	-	8.49	-
2471.9	4.7	-	6.5
2472	-	5.76	-
2473.2	8.9	1.69	5.3
2474.4	5.5	-	7.6
2479.2	-	0.62	-
2490	-	0.22	-

**Table 2:** Charge transfer times ($\tau_{ct}$) in femtoseconds (fs) for MoS_2_/SiO_2_,P3HT/SiO_2_ and P3HT/MoS_2_/SiO_2_ [51].

Photon Energy (eV)	$\tau_{ct}$ ***(fs)***			
			P3HT/MoS_2_/SiO_2_	
	MoS_2_/SiO_2_	P3HT/SiO_2_	MoS_2_	P3HT
2470.5	1.32	-	1.25	-
2472.1	0.62	11.3	0.34	2.41
2472.9	0.5	4.13	0.2	0.45
2474.5	-	0.36	-	0.32

**Table 3:** Summary of the charge transfer times ($\tau_{ct}$) across P3HT-based heterojunctions measured by the CHC technique [48,51,56].

Material System	Charge Transfer Time (fs)	Reference
Pristine P3HT	4.7	[63]
P3HT/Fe-MWCNT-5%	6.5	[63]
P3HT:PCBM blend	0.22	[42]
P3HT/MoS_2_ (3p_z_)	0.34	[56]
P3HT/MoS_2_ ($\pi^{*}$(C-C))	0.45	[56]
